# The Preparation of W/O/W High-Internal-Phase Emulsions as Coagulants for Tofu: The Effect of the Addition of Soy Protein Isolate in the Internal Water Phase

**DOI:** 10.3390/foods13172748

**Published:** 2024-08-29

**Authors:** Yongquan Wang, Xuanbo Liu, Qiang Zhang

**Affiliations:** 1 Key Laboratory of Jianghuai Agricultural Product Fine Processing and Resource Utilization, Ministry of Agriculture and Rural Affairs, Anhui Engineering Research Center for High Value Utilization of Characteristic Agricultural Products, Food Processing Research Institute, Department of Food Science and Engineering, Anhui Agricultural University, Hefei 230036, China; wangyongquan@ahau.edu.cn; 2 Department of Food Science and Technology, Virginia Polytechnic Institute and State University, 1230 Washington Street SW, Blacksburg, VA 24061, USA; xuanbol@vt.edu

**Keywords:** W/O/W HIPE, soy protein isolate, stability, microstructure, tofu

## Abstract

Tofu quality is determined by a controlled coagulation process using a W/O/W emulsion coagulant. The impact of adding soy protein isolate (SPI) to the inner water phase on the stability of W/O/W high-internal-phase emulsions (HIPEs) and its application as a coagulant for tofu was assessed. No creaming occurred during 7-day storage with SPI concentrations up to 0.3%, while the emulsion droplets aggregated with 0.5% and 0.7% SPI. Emulsions containing 0.3% SPI maintained a constant mean droplet size after 21 days of storage and exhibited the lowest TURBISCAN stability index value. HIPE stability against freeze–thaw cycles improved after heating. HIPEs with SPI concentrations above 0.3% demonstrated an elastic gel-like behavior. The increased viscosity and aggregation of the protein around droplets indicated that the interaction among emulsion droplets could enhance stability. W/O/W HIPE coagulants significantly increased tofu yield, reduced hardness, and produced a more homogenous tofu gel compared to a MgCl_2_ solution. The HIPE with 0.3% SPI was found to be optimal for use as a coagulant for tofu.

## 1. Introduction

Tofu, also known as bean curd, is a traditional soy food that was invented in China during the Western Han Dynasty from 202 BC to 8 AD [1]. The consumption of tofu has increased globally, and it has high nutritional value and multiple health benefits [2]. It is considered one of the best plant-based protein sources due to its content of beneficial lipids, including linoleic and linolenic acids, and bioactive compounds such as isoflavones, saponins, and phytosterols. Traditional tofu manufacturing involves the heat treatment of soymilk, coagulation, and pressing [3]. Coagulation is the key step in converting soymilk to tofu curd. The quality and yield are determined by the coagulant type and concentration [4]. Tofu with different textures can be produced depending on the type of coagulant applied. Commonly used coagulants include glucono-δ-lactone (GDL), calcium sulfate (CaSO_4_), and magnesium chloride (MgCl_2_) [5,6]. Tofu produced with the application of MgCl_2_ retains a more natural flavor and taste of soybean and is preferred by consumers. However, the rapid solidification of soymilk via MgCl_2_ leads to a low yield of tofu with a coarse structure [7]. To control the coagulation process of soymilk, a novel coagulant was developed by the encapsulation of MgCl_2_ in water-in-oil (W/O) emulsion [8]. Tofu with a higher yield and better microstructure can be produced because W/O emulsion can control MgCl_2_ release [9]. However, the insolubility of W/O in water restricts its use in tofu processing. To address these problems, MgCl_2_ was encapsulated in a water-in-oil-in-water (W/O/W) emulsion and utilized as a tofu coagulant.

The W/O/W emulsion coagulant can be fabricated with a two-step method. First, MgCl_2_ is encapsulated in a primary W/O emulsion, which is stabilized by a hydrophobic emulsifier. Then, the W/O/W emulsion is formed by dispersing the primary W/O emulsion in an aqueous solution and stabilizing it with a hydrophilic emulsifier. Previous studies have utilized polyglycerol polyricinoleate (PGPR) as a hydrophobic emulsifier and decaglycerol monooleate (DGMO) as a hydrophilic emulsifier in the fabrication of W/O/W emulsion coagulants [10,11]. Given the consumer demand for green-label products, replacing synthetic emulsifiers with natural emulsifiers may be more attractive. Additionally, in the W/O emulsion, the mass ratio of the inner water phase containing MgCl₂ to the oil phase is 4:6. When this W/O emulsion is incorporated into a W/O/W emulsion, its proportion is either 50% or 60%. Consequently, the mass of MgCl₂ solution present in the W/O/W emulsion coagulant is less than 40% of that in the original solution. This will lead to requiring a large addition of emulsion coagulant to obtain the same MgCl_2_ concentration as the direct addition of a MgCl_2_ solution. W/O/W high-internal-phase emulsions (HIPEs) may help in reducing the usage amount of emulsion coagulant in tofu production by increasing the encapsulated MgCl_2_ concentration. 

Our previous research indicates that O/W HIPE can be fabricated with soy protein isolate (SPI) as the emulsifier at pH 3 [12]. The combination of O/W HIPE and W/O/W emulsions is expected to result in a HIPE consisting of double emulsions [13]. To the best of our knowledge, no information is available on W/O/W HIPE with SPI as a hydrophilic emulsifier. Furthermore, previous studies have suggested that the presence of bovine serum albumin and whey protein isolate in double emulsions can increase double emulsion stability [10,11,14]. The incorporation of SPI in double emulsions may also affect W/O/W emulsion stability. The objective of the present study is to fabricate W/O/W HIPE, explore the impact of the addition of SPI to the inner water phase on W/O/W HIPE stability, and investigate the application of W/O/W HIPE as a novel coagulant in tofu production.

## 2. Materials and Methods

### 2.1. Materials

Low-denatured and defatted soy flake (containing 48% protein) was acquired from Shandong Yuwang Ecological Food Industry Co., Ltd. (Dezhou, China). Nile red was obtained from Beijing Solarbio Science & Technology Co., Ltd. (Beijing, China). Nile blue A was acquired from Sigma-Aldrich Shanghai Trading Co Ltd. (Shanghai, China). Corn oil and PGPR were purchased from Shanghai Yuanye Biological Technology Co., Ltd. (Shanghai, China). MgCl_2_·6H_2_O was obtained from Lanyi Co., Ltd. (Beijing, China).

### 2.2. Preparation of Soy Protein Isolate (SPI)

The extraction of SPI from defatted soy flakes was processed with the method described in our previous research [12]. Defatted soy flakes were first milled and then passed through a 60-mesh sieve. The powder was dispersed in deionized water at a ratio of 1:10 (*w*/*v*), followed by adjusting to pH 8.0 with 2 M NaOH and stirring for 2 h at room temperature. The mixture was centrifuged at 9000× *g* for 20 min at 4 °C. The supernatant was collected, and the pH was adjusted to 4.5 using 2 M HCl, followed by centrifugation at 9000× *g* for 10 min at 4 °C. The precipitate was washed twice with deionized water and then neutralized to pH 7.5 with 2 M NaOH. The solution was dialyzed (8000 Da cut-off) against deionized water for 48 h at 4 °C and subsequently lyophilized. The protein content in the extracted SPI was 92% (dry basis), as measured by the Kjeldahl method [15].

### 2.3. Fabrication of W/O/W HIPE

The internal aqueous phase (W_1_) was prepared by dispersing SPI (0 to 0.7%, *w*/*w*) and MgCl_2_ (2 M) in deionized water. The external aqueous phase (W_2_) was prepared by dissolving SPI (2%, *w*/*v*) in deionized water. After adjusting them to pH 3 and stirring for 2 h, the two aqueous phases were stored at 4 °C overnight to hydrate. The oil phase (O) was prepared by dispersing PGPR (2%, *w*/*w*) in corn oil and incubating the solution at 65 °C for 15 min. The W_1_/O emulsions were fabricated according to the methods established by Zhu et al. [9], with minor modifications. The internal aqueous phase was homogenized with a 60% (*w*/*w*) oil phase using a T18 digital Ultra-Turrax homogenizer (IKA, Staufen, Germany) at 13,000 rpm for 2 min, followed by passing through an AH-NANO high-pressure homogenizer (ATS Engineering Ltd., Brampton, Canada) at 600 bar. The W/O/W emulsion was fabricated by homogenizing the external aqueous phase with 85% (*w*/*w*) W_1_/O emulsions at 13,000 rpm for 1 min.

### 2.4. Evaluation of the Stability

The stability of the HIPEs under long-term storage, heating, and freeze–thaw conditions was assessed using methods adapted from Peng and Tang [16]. To evaluate their long-term storage stability, the HIPEs were sealed in vials and stored at 4 °C for 21 days. Their thermal stability was investigated by sealing the HIPEs in vials, subjecting them to 85 °C for 30 min, and then cooled at room temperature for 2 h. Their freeze–thaw stability was examined by subjecting both the unheated and HIPEs (heated at 85 °C for 30 min) to freezing at −20 °C for 24 h, followed by storage at room temperature for 2 h. 

Emulsion stability was further analyzed using a TURBISCAN Tower (Formulaction, Toulouse, France), following the procedure described by Raikos [17]. A 20 mL sample of the W/O/W emulsions was placed in glass test bottles, which were then scanned at 25 °C for 24 h in the stability analyzer. The overall stability was quantified by the TURBISCAN stability index (TSI), as calculated by TURBISCAN software version 1.10.36.

### 2.5. Characterization of W/O/W HIPE

#### 2.5.1. Measurement of Droplet Size

The droplet size of the W/O/W emulsion was determined using a FlowSync laser particle size analyzer (Microtrac Inc., Montgomeryville, PA, USA). The refractive indices used were 1.473 for the corn oil droplets and 1.333 for the water. The droplet size was expressed as the volume-weighted mean diameter.

#### 2.5.2. Encapsulation Efficiency of Magnesium Chloride

Emulsions (3 g) were mixed with 100 mL of deionized water and stirred at 440 rpm. The conductivity was determined with a DDS-307A conductivity meter (INESA Scientific Instrument Co., Ltd., Shanghai, China). The concentration of unentrapped MgCl_2_ in the external water phase was calculated from a standard curve of MgCl_2_ in water. The encapsulation efficiency (EE) was calculated using the following equation:

EE(%) = (m_1_ − m_2_)/m_1_ (1)

where m_1_ represents the addition of MgCl_2_ in the internal water phase, and m_2_ represents the amount of MgCl_2_ that had seeped into the outer water phase.

#### 2.5.3. Rheological Behavior

The rheological properties of freshly prepared HIPEs were measured using a Physica MCR 301 rheometer (Anton Paar, Graz, Austria). The rheometer was equipped with a 50 mm-diameter parallel plate geometry, and the gap was set to 1.0 mm before the experiment. The temperature was maintained at 25 °C using a circulating system. The flow sweep test was performed within a shear rate of 0.01–100 1/s to measure the viscosity. The stress sweep test was conducted to identify the linear viscoelastic region. The oscillatory experiment was carried out at a strain of 0.1% (within the linear viscoelastic region) with a frequency of 0.1–10 Hz to determine the storage modulus (G′) and loss modulus (G″). 

#### 2.5.4. Microstructure Observation

Optical micrographs were obtained using a CX23 optical microscope (Olympus, Tokyo, Japan), which was equipped with a digital camera. Freshly prepared emulsions were diluted 10-fold and observed using a 100× oil immersion objective lens.

Confocal laser scanning microscopy (CLSM) images were obtained by a Leica TCS SP8 confocal laser scanning microscope (Leica Microsystems Inc., Mannheim, Germany), utilizing a 63× oil-immersion objective lens. Nile red (0.1 mg/mL) and Nile blue A (0.1 mg/mL) were initially dissolved in ethanol. Subsequently, 2 mL of HIPEs was stained with 0.1 mL of the dye mixture and placed on concave slides. After covering them with coverslips, images were collected by excitation at wavelengths of 488 nm and 633 nm.

### 2.6. Application of W/O/W HIPE as a Coagulant for Tofu

#### 2.6.1. Gelation Process Investigation

The gelation process of soymilk was investigated through rheological analysis. Dynamic oscillatory time sweep tests were conducted using a Physica MCR 301 rheometer (Anton Paar, Graz, Austria) equipped with a 50 mm-diameter parallel plate geometry. The gap was set to 1.0 mm before the experiment, and the temperature was maintained using a circulating system. Various W/O/W emulsions or 2 M MgCl_2_ solution coagulants were mixed with pre-heated soymilk. The samples were then transferred to the parallel plates, and silicon oil was applied to cover the exposed surface to prevent water loss. After the temperature reached 85 °C, the storage modulus and loss modulus were recorded at a strain of 1% (within the linear viscoelastic region) and a frequency of 1 Hz.

#### 2.6.2. Tofu Preparation

Tofu was prepared according to a previously published method [10,18], with some modifications. Soybeans were cleaned and soaked in tap water for 12 h at 25 °C, using a soybean-to-water weight ratio of 1:3.5. After soaking, the soybeans were drained and milled with tap water at a weight ratio of 1:6 using a grinder. The mixture was then filtered through a 100-mesh nylon cloth to remove the okara. The raw soymilk was heated and boiled for 20 min. Following this, 500 mL of soymilk was cooled to 85 °C and mixed with either 6.50 g of 2 M MgCl_2_ solution or 19.11 g of W/O/W emulsion coagulant. The mixtures were incubated in a water bath at 85 °C for 10 min, followed by gentle agitation and further incubation for 20 min. The tofu gel was then broken into pieces and placed in a mold (10 cm × 7 cm × 8 cm). After pressing with a weight of 2200 g for 30 min, the tofu was removed from the mold.

#### 2.6.3. Measurement of Tofu Yield and Tofu Water Content

To determine the tofu yield, the mass of tofu produced from 500 mL of soymilk was recorded. To assess the water content of the tofu, 2.0 g of freshly prepared tofu samples were heated at 105 °C until a constant mass was achieved. The water content was then calculated based on the difference in mass before and after drying.

#### 2.6.4. Water-Holding Capacity (WHC) Determination

The WHC was measured following a previously published method [19], with some modifications. Fresh tofu samples (2.0 g) from the central portion were cut using a stainless steel knife and placed on degreasing cotton in a centrifuge tube. Water loss was measured after centrifuging at 465× *g* for 10 min. The WHC was defined as the percentage of retained water mass relative to the initial water mass in the tofu samples.

#### 2.6.5. Texture Profile Analysis (TPA)

The texture of the tofu was determined using a double compression test on a TA.HD plus texture analyzer (Stable Micro Systems, Surrey, UK), equipped with a 36 mm-diameter cylinder probe. Samples (1.5 cm × 1.5 cm × 1.5 cm) were cut from the central portion of the fresh tofu and compressed to 30% of their original height at a speed of 20 mm/min twice. The hardness, cohesion, springiness, gumminess, and chewiness were calculated from the TPA curve using the associated software.

#### 2.6.6. Scanning Electron Microscopic Observation

The microstructure of the tofu was observed with a SU3500 scanning electron microscope (Hitachi, Tokyo, Japan) at an accelerating voltage of 15.0 kV. Sample preparation was conducted according to the method described by Lee and Kuo [20], with a minor modification. Fresh tofu samples (1.5 cm × 1.5 cm × 1.5 cm) were cut from the central portion, frozen in liquid nitrogen, and lyophilized. The dried tofu samples were mounted on specimen stubs using double-sided adhesive tape and sputter-coated with a gold layer before imaging.

### 2.7. Statistical Analysis

The data were analyzed using analysis of variance (ANOVA) followed by Duncan’s multiple comparison test. Differences were considered significant at *p* < 0.05. The graph was plotted with Origin version 9.1.0 (OriginLab Co., Northampton, MA, USA).

## 3. Results and Discussion

### 3.1. Storage Stability of W/O/W HIPE

**Figure 1:**
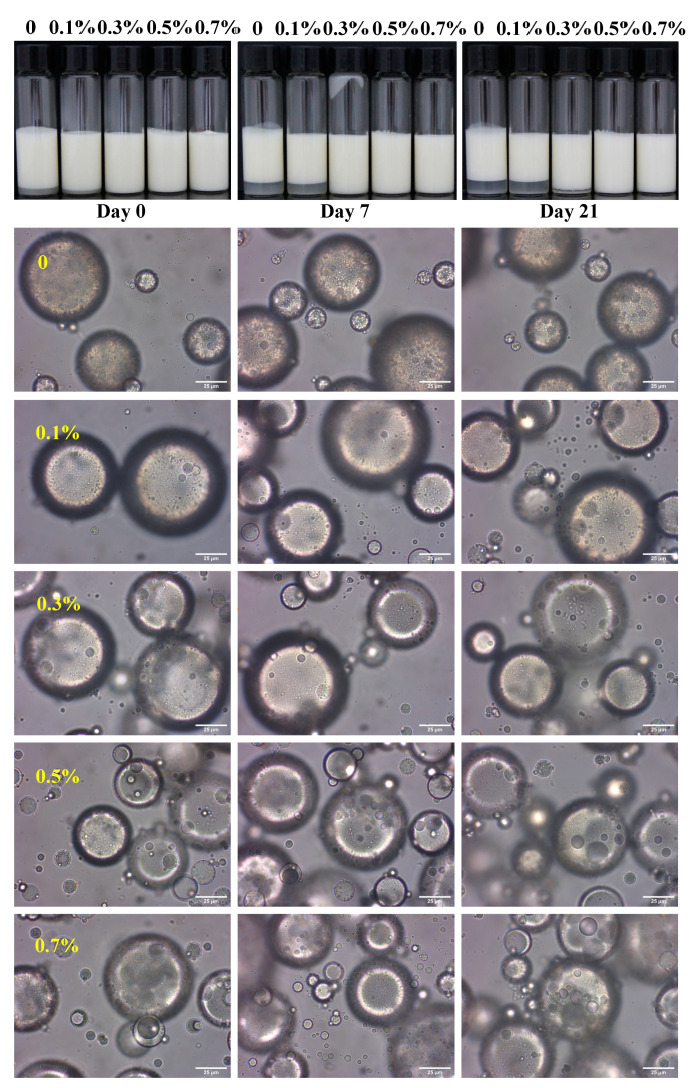
Photograph and optical micrograph of water-in-oil-in-water high-internal-phase emulsions (W/O/W HIPEs) with varied soy protein isolate (SPI) addition in the internal water phase after storage for 0, 7, and 21 days. Scale bars represent 25 μm.

The photographs and optical micrographs of the W/O/W HIPEs are presented in Figure 1, illustrating the influence of the SPI content in the internal water phase. The stability of the W/O/W emulsion was significantly enhanced with increasing SPI concentrations. At an SPI concentration of 0.3%, the emulsions remained stable during 7 days of storage. When the SPI concentration was increased to 0.5% and 0.7%, no creaming was observed even after 21 days of storage. These findings align with previous studies, where the addition of whey protein isolate or bovine serum albumin in the internal water phase significantly improved the stability of W/O/W emulsions [10]. The improved stability can be attributed to the interaction between PGPR and the co-adsorbed protein molecules at the inner interface, resulting in increased interfacial viscoelastic moduli [21]. Although creaming occurred in the W/O/W emulsions without or with 0.1%, and 0.3% SPI addition, no significant changes were observed in the microstructure. W/O droplets were visible in all the emulsions, indicating the successful preparation of W/O/W emulsions. However, as the SPI concentration increased to 0.5% and 0.7%, the W/O/W emulsion droplets tended to aggregate. This suggests that the addition of SPI in the internal water phase may lead to an increase in emulsion viscosity, which in turn prevents creaming. Additionally, the W/O emulsion droplets within the emulsions exhibited more significant growth after 7 and 21 days of storage. This may be attributed to swelling caused by the osmotic pressure between the two water phases [22].

**Figure 2:**
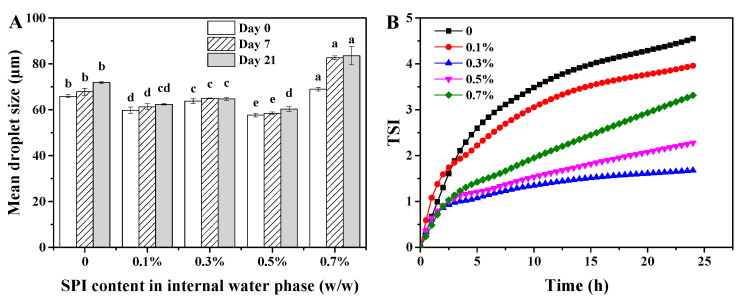
Properties of W/O/W HIPE with various SPI additions in internal water phase. (**A**) Mean droplet size after storage for 0, 7, and 21 days, (**B**) TURBISCAN stability index (TSI) value. Different letters (a–e) above bars after storage for the same days indicate significant differences (*p* < 0.05).

The mean droplet size of W/O/W emulsions as influenced by SPI addition and storage duration is depicted in Figure 2A. When SPI addition was raised to 0.5%, the mean droplet size of W/O/W emulsions was notably decreased from 65.91 nm to 57.67 nm. This size reduction can be attributed to the enhanced stability of the emulsions, which is likely due to the interaction between PGPR and protein molecules [10]. However, with a further increase in the SPI concentration to 0.7%, the mean droplet size increased to 68.98 nm. This enlargement may be explained by a reduction in the interfacial elasticity modulus of the film formed by the mixture of PGPR and protein as the protein concentration exceeded 0.5% [11]. After 21 days of storage, the droplet size of the control samples increased significantly to 71.89 nm. A similar increase was observed in the W/O/W emulsions with 0.1%, 0.5%, and 0.7% SPI additions, with the droplet size of the 0.7% SPI emulsion even increasing after just 7 days of storage. In contrast, the mean droplet size of the W/O/W emulsions with a 0.3% SPI addition showed no significant change after 21 days of storage. 

The stability of W/O/W emulsions was further assessed by multiple light scattering, and the TURBISCAN stability index (TSI) was calculated. As illustrated in Figure 2B, the increase in TSI values across all emulsions after 24 hours signifies a general trend of emulsion instability. However, the incorporation of SPI in the internal water phase markedly reduced the TSI values compared to the emulsions without SPI. This decrease in the TSI aligns with previous findings, where the addition of gelatin to the inner water phase of W/O/W emulsions similarly reduced the TSI values [23]. Notably, as the SPI concentration increased to 0.3%, the TSI values decreased evidently. However, further increasing the SPI concentration from 0.3% to 0.5% and 0.7% led to a rise in the TSI values. The lowest TSI values, observed at 0.3% SPI addition, suggest that these emulsions possessed the relatively highest stability.

The creaming and increase in droplet size of the W/O/W emulsions with the addition of 0.1% SPI implies that an SPI below 0.1% was not enough to improve the emulsion stability. Conversely, the incorporation of SPI at concentrations exceeding 0.5% appeared to induce emulsion instability. This was proven by the increase in the W/O emulsion droplets inside emulsions and droplet size after storage for 21 days. These observations are further supported by the findings that emulsions containing 0.3% SPI exhibited the lowest TSI values. Consequently, the inclusion of 0.3% SPI in the inner water phase may be optimal for enhancing the storage stability of W/O/W emulsions.

### 3.2. Freeze–Thaw Stability of W/O/W Emulsions

**Figure 3:**
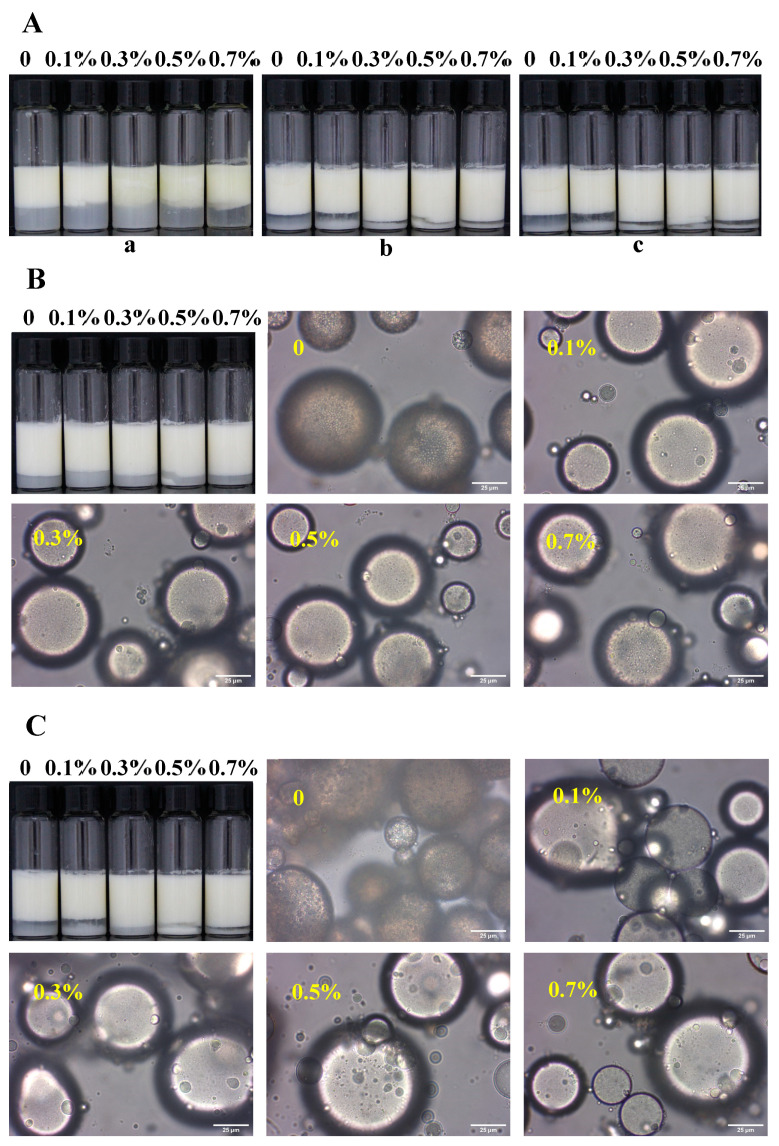
Photographs and optical micrographs of W/O/W HIPE with various SPI additions in the internal water phase. (**A**) Photographs of unheated HIPEs subjected to freeze–thawing once (**a**), heated HIPEs subjected to freeze–thawing twice (**b**) and thrice (**c**). (**B**) Photographs and optical micrographs of heated HIPEs. (**C**) Photographs and optical micrographs of heated HIPEs after freeze–thawing once. Scale bars represent 25 μm.

All the unheated W/O/W emulsions exhibited phase separation after freeze–thaw, as displayed in Figure 3A. The extent of oil leakage increased with the addition of SPI to the inner water phase. Our previous research demonstrated that heating significantly improved the freeze–thaw stability of O/W HIPEs stabilized by SPI through interactions between SPI and the emulsions [12]. To determine if heating could similarly enhance the freeze–thaw stability of W/O/W emulsions, the emulsions were heated at 85 °C for 30 min. Although all heated emulsions underwent creaming, no oil leakage was observed, as illustrated in Figure 3B. Microstructural analysis revealed that freeze–thawing induced swelling of the W/O emulsion droplets within the heated W/O/W emulsions. However, no oil was released from the heated emulsions after the freeze–thaw treatment, indicating that heat treatment significantly enhanced the freeze–thaw stability of W/O/W emulsions, as displayed in Figure 3C. The heated W/O/W emulsions can even suffer freeze–thawing thrice without oil leaking, as illustrated in Figure 3A. The enhanced stability of the heated emulsions can be attributed to the aggregation of protein particles around the droplets. Furthermore, the volume of the separated water phase in emulsions with 0.3–0.7% SPI addition was notably less than that in emulsions without SPI or with 0.1% SPI addition, consistent with the observed storage stability of the W/O/W emulsions. These results further confirm that the stability of W/O/W emulsions is enhanced by the addition of SPI to the inner water phase.

### 3.3. Rheological Behavior of W/O/W Emulsions

**Figure 4:**
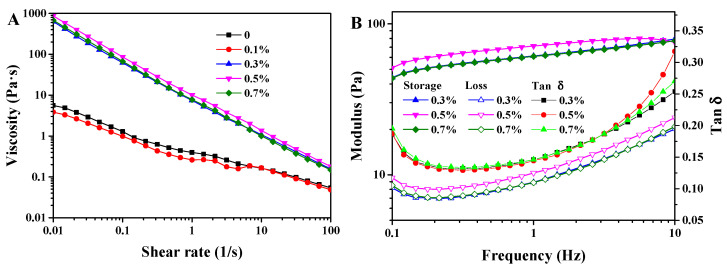
Rheological behavior of W/O/W HIPE with various SPI additions in the internal water phase. (**A**) Viscosity. (**B**) Storage, loss modulus, and tan δ.

**Figure 5:**
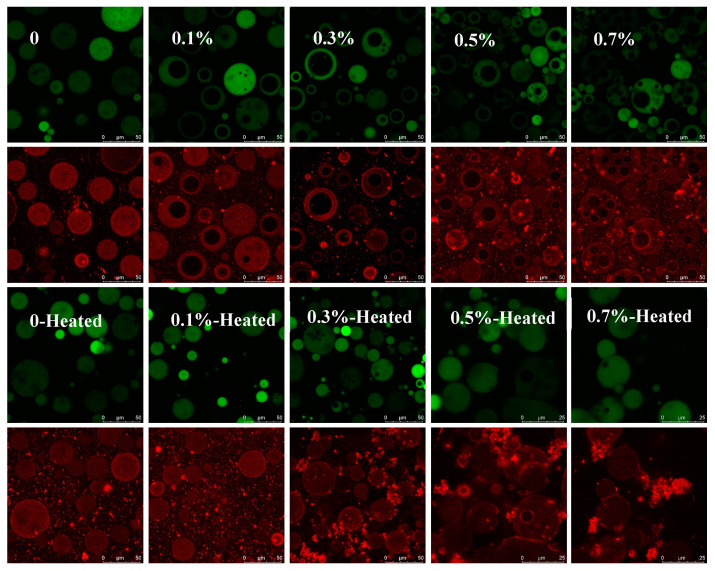
Confocal laser scanning microscopy images of unheated and heated W/O/W HIPEs with various SPI additions in the internal water phase. The emulsions were stained with both Nile blue A and Nile red, which visualized the oil phase in green and the protein in red. Scale bars are 25 μm.

The rheological behavior of emulsions with SPI concentrations ranging from 0 to 0.7% in the internal water phase was assessed. As shown in Figure 4A, all emulsions exhibited shear-thinning behavior, with viscosity decreasing as the shear rate increased. In addition, the viscosity of emulsions with 0.1% SPI was similar to that of the emulsions without SPI, whereas emulsions containing 0.3% to 0.7% SPI in the internal water phase exhibited significantly higher viscosities. This observation is consistent with the absence of creaming in emulsions with 0.3% to 0.7% SPI after 7 days of storage, whereas creaming was observed in emulsions without SPI or with 0.1% SPI. The increased viscosity of these emulsions can be attributed to the migration of water between the two aqueous phases [24]. Higher SPI concentrations in the internal phase facilitated the transfer of water from the external aqueous phase, which is consistent with the observed increase in the size of the W/O emulsion droplets within the W/O/W emulsions after storage. Aggregation can be observed in the micrographs of the W/O/W HIPEs (Figure 1), which likely contributed to the increased viscosity observed with higher SPI concentrations. A similar phenomenon has been reported in emulsions stabilized by whey protein aggregates, where an increase in protein aggregate size was associated with higher emulsion viscosity [25]. Furthermore, the aggregation of protein particles around the droplets became more pronounced with the increasing SPI concentration (Figure 5), which also contributed to the elevated viscosity of the emulsion.

The storage modulus (G′), loss modulus (G″), and tan δ (tan δ = G″/G′) are depicted in Figure 4B. It is worth noting that the emulsions with or without a 0.1% SPI addition in the inner water phase were in a liquid state, and therefore, were not subjected to oscillatory experiments. The emulsions with 0.3% to 0.7% SPI possessed higher G′ values than G″ values, exhibiting a predominantly elastic gel-like behavior [26]. This behavior is consistent with that observed in the gel-like O/W HIPEs, which also showed higher G′ values relative to G″ [12]. The G′ values rose with the increasing frequency, indicating that the gel-like network may be formed by non-covalent physical crosslinking [27]. Given the liquid state of emulsions without SPI or with a 0.1% SPI addition, the formation of the gel-like network is likely attributed to the non-covalent interactions among SPI aggregates. This is in agreement with the emulsion viscosity and protein aggregations presented around the droplets. The increased values of tan δ at a high frequency indicate weakened solid properties [28,29], further supporting the role of non-covalent interactions. Emulsions with 0.3% SPI in the inner water phase exhibited lower tan δ values compared to those with 0.5% and 0.7% SPI, signifying enhanced solid-like characteristics.

### 3.4. Microstructure of W/O/W Emulsions

The confocal laser scanning microscopy images of the W/O/W emulsions are displayed in Figure 5. The micrographs of the oil phase show that the emulsion droplets tended to aggregate with increasing additions of SPI in the inner water phase. This aggregation was further pronounced following the heat treatment, which led to the formation of protein aggregates surrounding the droplets. This is consistent with our previous study showing that heat treatment promoted SPI interaction due to the increased surface hydrophobicity [12]. As the SPI concentration increased, a greater amount of protein accumulated around the droplets, and the heat treatment intensified this aggregation. When the SPI addition exceeded 0.3%, the protein aggregates facilitated connections among the droplets, contributing to significant increases in the emulsion viscosity and stability for emulsions containing 0.3% to 0.7% SPI.

### 3.5. Application of W/O/W HIPE as a Coagulant for Tofu

**Figure 6:**
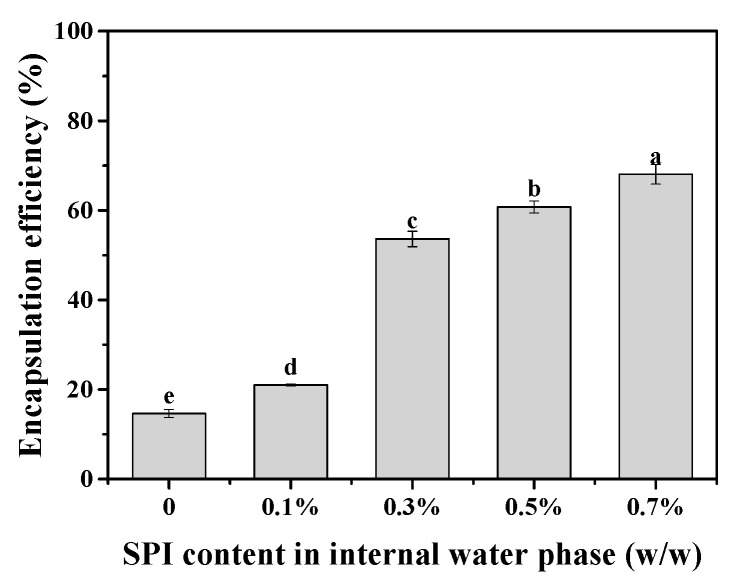
Encapsulation efficiency of MgCl_2_ in W/O/W HIPE with various SPI additions in the internal water phase. Different letters (a–e) above bars indicate a significant difference (*p* < 0.05).

Figure 6 illustrates the encapsulation efficiency (EE) of MgCl₂ in W/O/W HIPEs with varying SPI concentrations. The SPI addition in the inner water phase significantly increased the EE of MgCl_2_ in the W/O/W HIPE. The EE values increased from 14.65% to 68.05% with SPI incorporation. Furthermore, the addition of 0.1% SPI resulted in an EE increase of 6.35%, whereas SPI concentrations of 0.3% to 0.7% led to a more pronounced rise in EE, ranging from 38.98% to 53.40%. These results suggest that MgCl₂ can be effectively encapsulated within W/O/W HIPE, highlighting the potential of HIPE as a coagulant for tofu production. The variation in the SPI content may influence tofu gelation by modifying the EE of MgCl₂ in the W/O/W HIPE system. 

**Figure 7:**
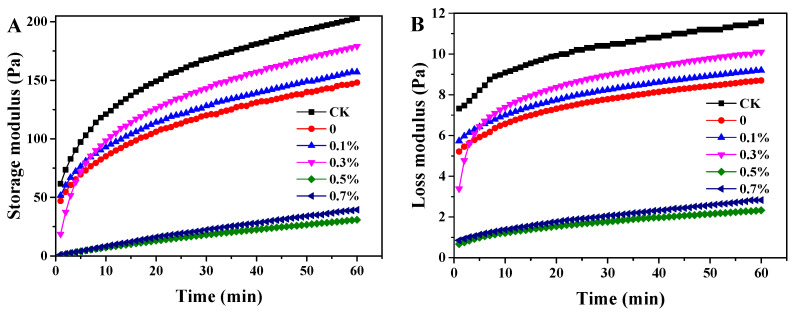
(**A**) Storage modulus and (**B**) loss modulus of soymilk heated at 85 °C over 60 min with different coagulants.

The gelation process of tofu using either MgCl₂ or W/O/W emulsion coagulants was simulated by analyzing the rheological behavior. The G′ and G″ of soymilk heated at 85 °C over 60 min with different coagulants were measured and are depicted in Figure 7. Both the G′ and G″ values increased rapidly within the first 10 min for all samples. Furthermore, the G′ values were consistently higher than the G″ values, indicating that the gel point was reached upon mixing the coagulants with pre-heated soymilk [9]. Notably, the G′ and G″ values for soymilk gelled with MgCl₂ coagulant were significantly higher compared to those for the W/O/W emulsion coagulants. This suggests that the encapsulation of MgCl₂ within the W/O/W emulsions may slow the gelation process of soymilk. The G′ and G″ values varied significantly for the soymilk gelled with W/O/W emulsion coagulants containing different concentrations of SPI in the inner water phase. Specifically, samples with 0.5% and 0.7% SPI exhibited the lowest G′ and G″ values. This can be attributed to the higher encapsulation efficiency and viscosity associated with W/O/W emulsions containing these SPI concentrations. Conversely, the G′ and G″ values for the samples with 0.1% SPI were notably higher than those for the samples without SPI addition. Interestingly, the samples with 0.3% SPI initially showed lower G′ and G″ values compared to those without SPI, but these values increased to exceed those observed for the samples with 0.1% SPI within the first 10 min. It is worth noting that the upper soymilk gelled within the first 10 min after mixing it with the W/O/W emulsions with a 0.3% SPI addition. This phenomenon likely resulted from the tendency of the W/O/W emulsions to rise to the upper layer of the soymilk upon mixing, thereby inducing gelation around the emulsions. This observation is consistent with the preparation of tofu and explains the initial rapid increase in the G′ and G″ values and the subsequently higher values recorded. 

The yield, moisture, WHC, and texture of the freshly prepared tofu are summarized in Table 1. Significant differences in yield were observed between the tofu prepared with MgCl₂ and that made with W/O/W HIPE. The tofu produced using W/O/W HIPE demonstrated a higher yield compared to the tofu made with MgCl₂, which yielded 167.20 g. Specifically, the yield of tofu increased from 201.53 g to 233.78 g with the addition of SPI in the inner water phase of W/O/W HIPE, reaching a maximum of 0.7% SPI. In contrast, the yield of tofu was comparable between formulations without SPI and those with 0.3% SPI. These variations in yield correspond with changes in the moisture content, consistent with previous studies that have shown a strong correlation between yield and moisture content in tofu production [4]. The WHC, which represents the amount of water retained within the tofu gel network, was highest in the tofu prepared with MgCl₂, at 58.76%. This value was notably higher compared to the tofu produced with W/O/W emulsions containing 0.5% and 0.7% SPI. The reduced WHC in these emulsions can be attributed to the less robust gel network, which is more susceptible to deformation under centrifugation [30]. The texture of tofu is a critical factor influencing product acceptability. The tofu prepared with MgCl₂ exhibited the highest values for hardness, gumminess, and chewiness, indicating that it achieved a firmer consistency. In comparison, the tofu prepared with W/O/W emulsion coagulants demonstrated lower hardness. This reduction in hardness is likely due to the slower coagulation process associated with emulsions, which results in a gel network that retains more water [7]. Consequently, less force is required to compress the tofu, leading to a softer texture [31]. Cohesion reflects the degree to which the tofu can be compressed before breaking, while springiness measures its ability to return to its original length after stretching. No significant differences in the cohesion or springiness were observed between the tofu coagulated with MgCl₂ and those using emulsions with SPI concentrations ranging from 0% to 0.5%. However, the tofu coagulated with emulsion containing 0.7% SPI showed decreases in both cohesion and springiness. These reductions indicate that the gel network might be compromised due to the slower solidification process associated with higher SPI concentrations. Gumminess and chewiness are derived by multiplying the hardness by the cohesiveness and the gumminess by the springiness, respectively. The observed decreases in the gumminess and chewiness are consistent with the changes in hardness for the tofu prepared with emulsion coagulants.

**Figure 8:**
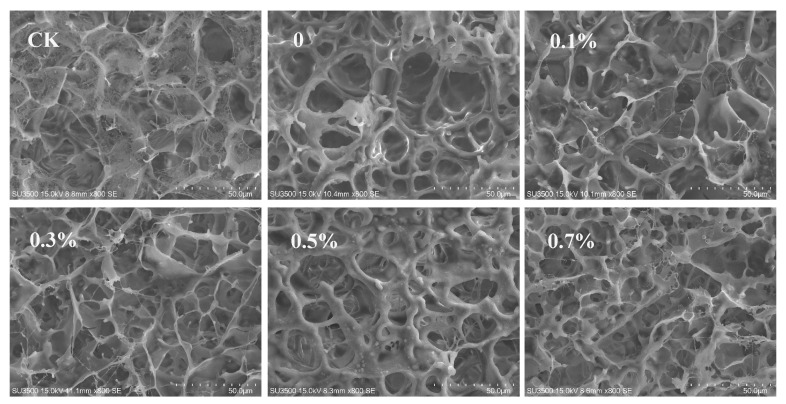
Scanning electron microscopy micrographs of tofu prepared with different coagulants. Scale bars are 50 μm.

The microstructure of the tofu prepared with MgCl₂ and various emulsion coagulants is presented in Figure 8. All tofu samples displayed a honeycomb-like structure, consistent with previous studies [20]. The control tofu exhibited a rough and loose network with relatively large pores. In contrast, the tofu coagulated with the W/O/W emulsions demonstrated a more homogeneous network with deeper pores. These micropores may better retain water, contributing to the higher moisture content observed in the tofu prepared with emulsion coagulants. A slower gelation rate of denatured soy proteins aids in the formation of a more filamentous network [32]. Increasing the SPI concentration in the inner water phase of the emulsion coagulants led to greater interconnection of the protein backbones and the formation of smaller pores.

## 4. Conclusions

A W/O/W HIPE containing MgCl₂ was successfully prepared, with its storage stability significantly enhanced by the addition of SPI in the inner water phase. The HIPE with a 0.3% SPI addition possessed the highest stability, as indicated by the unchanged mean droplet size and the lowest TSI values after storage. Heat treatment significantly improved the freeze–thaw stability of the HIPE, allowing it to withstand up to three freeze–thaw cycles without oil leakage. This enhanced stability is attributed to increased interactions among the emulsion droplets. The HIPE with 0.3% SPI exhibited lower tan δ values compared to those with 0.5% and 0.7% SPI, indicating stronger solid-like properties. CLSM images confirm the protein particle aggregation around droplets, reinforcing the interaction of emulsion droplets. The addition of SPI in the inner water phase resulted in a significant increase in the tofu yield, a decrease in the tofu hardness, and a more homogeneous gel structure. The HIPE with 0.3% SPI demonstrated superior stability and significantly enhanced the MgCl₂ encapsulation efficiency, making it a suitable coagulant for tofu production. This research suggests that SPI can serve as an effective hydrophilic emulsifier and stabilizer in W/O/W HIPE applications. Future research will focus on exploring the nutritional changes and health benefits associated with HIPE coagulants in tofu production.

## Figures and Tables

**Table 1:** Yield, moisture, WHC, and texture of tofu prepared with different coagulants.

Coagulant	CK	0	0.1%	0.3%	0.5%	0.7%
Yield (g/500 mL)	167.20 ± 9.71 ^d^	201.53 ± 4.31 ^bc^	187.21 ± 9.76 ^cd^	201.84 ± 6.86 ^bc^	222.17 ± 18.80 ^ab^	233.78 ± 16.14 ^a^
Moisture (%)	77.91 ± 0.22 ^d^	79.16 ± 0.48 ^bc^	78.03 ± 1.05 ^d^	78.22 ± 0.43 ^cd^	80.09 ± 0.71 ^b^	81.85 ± 0.35 ^a^
WHC (%)	58.76 ± 5.03 ^a^	55.18 ± 3.08 ^ab^	58.53 ± 3.85 ^a^	58.44 ± 1.02 ^a^	50.31 ± 2.70 ^b^	50.06 ± 4.42 ^b^
Hardness	211.02 ± 8.39 ^a^	86.02 ± 1.64 ^e^	138.94 ± 6.92 ^b^	112.76 ± 9.18 ^c^	101.48 ± 1.89 ^d^	88.06 ± 4.12 ^e^
Cohesion	0.81 ± 0.01 ^a^	0.80 ± 0.02 ^a^	0.81 ± 0.01 ^a^	0.79 ± 0.01 ^ab^	0.79 ± 0.02 ^ab^	0.76 ± 0.01 ^b^
Springiness (%)	93.25 ± 0.42 ^ab^	91.91 ± 3.12 ^ab^	93.52 ± 0.72 ^ab^	94.10 ± 0.54 ^a^	93.32 ± 0.32 ^ab^	91.12 ± 0.67 ^b^
Gumminess	170.40 ± 4.12 ^a^	68.57 ± 1.25 ^e^	112.24 ± 6.52 ^b^	88.97 ± 8.33 ^c^	79.83 ± 1.33 ^d^	67.17 ± 2.94 ^e^
Chewiness	158.89 ± 3.15 ^a^	63.02 ± 2.07 ^e^	104.98 ± 6.63 ^b^	83.70 ± 7.44 ^c^	74.49 ± 1.25 ^d^	61.20 ± 2.42 ^e^

Different letters (a–e) in the same line indicate a significant difference (*p* < 0.05).

## Data Availability

The original contributions presented in the study are included in the article. Further inquiries can be directed to the corresponding author.
